# Applied microbiology of the phyllosphere

**DOI:** 10.1007/s00253-024-13042-4

**Published:** 2024-02-15

**Authors:** Lorena I. Rangel, Johan H. J. Leveau

**Affiliations:** 1 Cell & Molecular Sciences, The James Hutton Institute, Dundee, Scotland, UK; 2 Department of Plant Pathology, University of California, Davis, CA USA

**Keywords:** Bioprospecting, Biomonitoring, Bioprocessing, Biosupplementing, Phylloremediation, Phyllosphere

## Abstract

**Abstract:**

The phyllosphere, or plant leaf surface, represents a microbial ecosystem of considerable size, holding extraordinary biodiversity and enormous potential for the discovery of new products, tools, and applications in biotechnology, agriculture, medicine, and elsewhere. This mini-review highlights the applied microbiology of the phyllosphere as an original field of study concerning itself with the genes, gene products, natural compounds, and traits that underlie phyllosphere-specific adaptations and services that have commercial and economic value for current or future innovation. Examples include plant-growth-promoting and disease-suppressive phyllobacteria, probiotics and fermented foods that support human health, as well as microbials that remedy foliar contamination with airborne pollutants, residual pesticides, or plastics. Phyllosphere microbes promote plant biomass conversion into compost, renewable energy, animal feed, or fiber. They produce foodstuffs such as thickening agents and sugar substitutes, industrial-grade biosurfactants, novel antibiotics and cancer drugs, as well as enzymes used as food additives or freezing agents. Furthermore, new developments in DNA sequence-based profiling of leaf-associated microbial communities allow for surveillance approaches in the context of food safety and security, for example, to detect enteric human pathogens on leafy greens, predict plant disease outbreaks, and intercept plant pathogens and pests on internationally traded goods.

**Key points:**

• *Applied phyllosphere microbiology concerns leaf-specific adaptations for economic value*

• *Phyllobioprospecting searches the phyllosphere microbiome for product development*

• *Phyllobiomonitoring tracks phyllosphere microbial profiles for early risk detection*

**Graphical Abstract:**

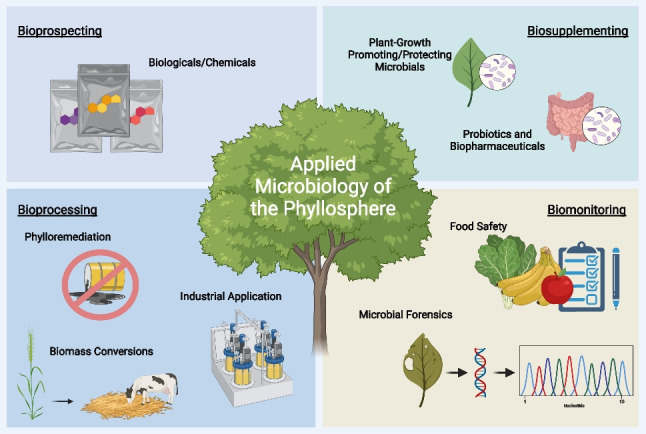

## Introduction

Plant foliage is one of the largest and most important resources sustaining life on our planet. Terrestrial vegetation across both hemispheres represents an estimated leaf mass of 30 Gt and a surface area that is twice that of the land mass surface (Bar-On and Milo 2019). Plant leaves provide many services to humans and other life on Earth. They are a source of oxygen, pharmaceuticals (e.g., artemisinin or baccatins, which are precursors to the cancer drug paclitaxel), and recreational drugs (e.g., nicotine, cannabis); they offer shade and landscape aesthetics; serve as nourishment (e.g., leafy greens), drink (e.g., tea), or animal feed (e.g., grass-fed cows); and support through their photosynthetic properties the production of plant-based foods (e.g., fruits, nuts, and vegetables) and other products (e.g., fiber, fuel, lumber, and paper). An underappreciated but crucial source of many other services associated with plant foliage is the rich and diverse microbial communities that plants carry on their leaf surfaces (Bashir et al. 2022). In this review, we will explore these communities in detail, with a particular focus on how the properties, genes, and gene products of these leaf surface microbiota may be applied and exploited for improving plant, human, and environmental health. The novelty of this review lies in the focused and specialized synthesis of the current literature to highlight an active and exciting field of study known as applied microbiology of the phyllosphere, which is presented here in terms of bioprospecting, biosupplementing, bioprocessing, and biomonitoring.

## Microbial traits and adaptations to survive and thrive in the leaf surface environment

As a habitat for microorganisms, plant leaves are referred to as the phyllosphere (Koskella 2020; Leveau et al. 2023a; Sohrabi et al. 2023). Microorganisms inhabiting aerial leaf surfaces include bacteria, fungi, yeasts, protists, archaea, and viruses. State-of-the-art DNA-based community profiling techniques have revealed an enormous diversity in the types and functions of leaf surface colonizers (Fadiji and Babalola 2020). Bacteria and filamentous fungi are among the most researched members of the phyllosphere, but the number of studies related to community composition of other microbial groups, for example, yeasts (Gouka et al. 2022), protists (Taerum et al. 2023) and viruses (ter Horst et al. 2023), is on the rise. DNA-based approaches have also revealed that only a fraction of the microbes that can be recovered from leaf surfaces can be cultured in the lab (Müller and Ruppel 2014). In terms of numbers, bacteria are by far the most abundant colonizers of leaf surfaces and can typically be found at densities between 10^6^ and 10^8^ cells/cm^2^ (Andrews and Harris 2000; Hirano and Upper 2000). Microorganisms that survive and multiply on aerial plant surfaces are called residual (as opposed to transient) epiphytes and are often labeled as pathogens, saprophytes, beneficials, or commensals, depending on the outcome of the interactions that they have with their plant host. Relative abundances of these bacterial epiphytes in the phyllosphere are impacted by a large number of factors such as plant genotype and health, leaf age, environmental conditions, plant neighborhood, geographical location, and the interactions that the epiphytes have among themselves (Bao et al. 2020; Beilsmith et al. 2019; Kumar et al. 2019; Lajoie and Kembel 2021; Meyer et al. 2022; Mina et al. 2020; Schlechter et al. 2019; Shakir et al. 2021; Zhang et al. 2019). The use of gnotobiotic environments for plant growth has allowed a better understanding of the factors shaping phyllosphere microbial communities, including leaf genotype (Schäfer et al. 2022), phytopathogenesis (Vogel et al. 2021), and abiotic stresses (Molina et al. 2020; Yuan et al. 2016).

The leaf surface is considered a harsh environment for microorganisms, as it poses numerous challenges to survival and reproductive success. Many members of the phyllosphere-associated microbiota have acquired and evolved specific adaptations to deal with stresses that are common on the leaf surface, including low nutrient availability, exposure to ultraviolet (UV) light, desiccation, and fluctuating temperatures (Leveau 2019). Such adaptations often represent strategies of “tolerance” (Beattie and Lindow 1995) such as the synthesis of pigments to reduce the effects of harmful ultraviolet radiation (Jacobs et al. 2005; Kumar et al. 2016) and the production of protective biofilms to prevent desiccation (Arun et al. 2020). The latter adaptation is an example of “niche construction” (Manching et al. 2017) or “ecosystem engineering” where microbes change their local environment to increase their chances of survival (Baquero et al. 2021). Other examples involve the secretion of enzymes (Rocky-Salimi et al. 2016) or plant hormones such as indole 3-acetic acid or IAA (Vanderhoef and Dute 1981) to access nutrients, biosurfactants to enhance surface mobility (Nogales et al. 2015), or ice nucleation proteins which not only raise the temperature of freezing thereby breaking plant cells to release nutrients (Avalos-Ruiz et al. 2022) but also contribute to microbial airborne movement between plants (Maki et al. 2023).

Microbial survival in the phyllosphere not only relies on coping with the harsh conditions of a leaf surface but also requires interaction with other cohabitating microorganisms. The multitude of interactions that members of the leaf microbiota have with each other are key to shaping microbial community structure in the phyllosphere (Chaudhry et al. 2021; Rangel et al. 2021). Examples of such interactions are competition for nutrients and space (Schlechter et al. 2023), antibiosis through the production of lytic compounds, enzymes, secondary metabolites, and toxins (Qi et al. 2021; Rangel and Bolton 2022), or signal interference (e.g., quorum quenching) (Ma et al. 2013; Theodora et al. 2019). Each one of these interactions can by itself or in combination with others influence the growth, suppression, or even death of microorganisms inhabiting the same leaf or section of the leaf (Remus-Emsermann and Schlechter 2018). It has been demonstrated that early arrivers to the leaf surface can exert a so-called priority effect and outcompete immigrating microbes (Carlström et al. 2019), thus impacting ecological succession through niche pre-emption (Maignien et al. 2014). However, the survival of these keystone species is also affected by the growth stage of the host, with the persistence of core members contingent on core functions that are adapted for success in the leaf environment (Bell et al. 2021; Müller et al. 2016b). Another type of interaction in the phyllosphere is facilitation: examples include the promotion of fitness of bacterial foliar pathogens through intra-species communication (Li et al. 2019a) and the increased survival of bacterial species through inter-species signaling (He et al. 2022; Li and Tian 2012).

A powerful demonstration of the uniqueness of the leaf surface as a microbial habitat and of the specific microbial adaptations that allow life on the leaf surface comes from the comparative genomics analysis of two closely related foliar bacterial pathogens (Feil et al. 2005): *Pseudomonas syringae* pv. *syringae* B728a, which is a highly competent epiphyte and *Pseudomonas syringae* pv. *tomato* DC3000, which does not survive the leaf surface very well and prefers an endophytic lifestyle (i.e., inside the leaf tissue). It was shown that many of the genes and gene products unique to B728a are known to contribute to epiphytic fitness or “epiphytness” (Leveau et al. 2023b), such as the ice nucleation protein, an enzymatic pathway for the production of IAA, and genes for antibiosis and repair of UV-damaged DNA (Feil et al. 2005).

Seeing that the phyllosphere is a unique microbial habitat that requires a specific set of microbial adaptations and traits to increase chances of survival in the face of a range of biotic and abiotic challenges, we can expect that foliar microbial communities harbor species, strains, genes, and gene functions that have potential to be exploited for the discovery, development, and/or commercialization of novel products (Thapa and Prasanna 2018). Here, we provide an overview of functions that phyllosphere inhabitants are known to possess and that may be harnessed into products, processes, and technologies (Table 1). We will cover how the approach of bioprospecting (Müller et al. 2016a) contributes to the discovery of phyllosphere-derived microorganisms, enzymes, and metabolites with useful properties. We will also touch upon the use of biomonitoring, as it has been applied to other microbial habitats (Meyer et al. 2023; Michán et al. 2021), defined here as the practice of extracting information from microbial communities on leaf surfaces for the purpose of assessment and decision-making in the face of pathogen threats or other foliar risks.

**Table 1:** Applied phyllosphere services and overview of processes, technology, and product deliverables

Biotechnology concept	Application	Phyllosphere service	Engineering process	Phyllosphere-associated deliverables	Keywords	Selected experimental papers	Selected review papers
Bioprospecting			Mining for genes, natural compounds, and whole organisms in nature that have commercial economic value		Metagenomics, metaproteogenomics, natural products, and microbial diversity	Mishra et al. (2021); Singh et al. (2021)	Müller et al. (2016a)
	Biosupplementing						
		Plant health	Formulation of microbial cells to promote healthy phyllosphere growth	Nitrogen fixation, ACC deaminase, Plant-growth promoting phyllobacteria (PGPP)	Growth-promoting bacteria, probiotics, nitrogen fixation, biofertilizers, phytohormones, and plant-microorganism interactions	Abadi et al. (2021); Chacón et al. 2022); Ehau-Taumaunu and Hockett (2023); Minchev et al. (2021); Li et al. (2022); Stanton et al. (2019)	Berlec (2012); Hawkes et al. (2021)
		Human health	Formulation of microbial cells to promote human health	Lactic acid bacteria (LAB)	Edible microbiome, probiotics, lactic acid bacteria, gut microbiota, fermentation, raw foods, biofortification, and natural vaccination	Soto-Giron et al. (2021); Wassermann et al. (2017); Wicaksono et al. (2023a); Vitali et al. (2012)	Berg et al. (2014a); Ercolini and Fogliano (2018); Leeming et al. (2021); Marco et al. (2017); Roselli et al. (2021)
	Bioprocessing						
		Phylloremediation	Application of leaf-associated microbes for decontamination of polluted environments	Removal of airborne microplastics, pollutants, or pesticides	Plant-microorganism interactions, volatile organic compounds (VOCs), bioremediation, biodegradation, bioaugmentation, phyllopriming, and biotransformation	Ali et al. (2015); Crombie et al. (2018); Dharmasiri et al. (2023); Imperato et al. (2021); Jindachot et al. (2018); Kucharska et al. (2020)	Adomako and Yu (2023); Bringel and Couee (2015); Molina et al. (2021); Weyens et al. (2015); Wei et al. (2017)
		Biomass conversion	Microbial processing of organic materials by changing the chemical composition into a desired end product	Biofuels, silage, and curing	Biofuel production, bioaugmentation, and bioenergy	Ayala-Campos et al. (2022); Katsoula et al. (2020); Law et al. (2020); Miao et al. (2020); Miftah et al. (2022), Zhou et al. (2021)	García-Depraect et al. 2021; Rai et al. 2022; Sen et al. 2016; Zhalnina et al. 2021
		Industrial application	Large-scale production of chemical goods derived from microorganisms	Keltro, Xantural, SnoMax, Finase, Natuphos, Allzyme, and Bactroban	Cosmetics, silage, tobacco, pharmaceuticals, textiles, batch fermentation, and biosurfactants	Kraut-Cohen et al. (2016); Pokharel et al. (2021); Wex et al. (2015)	Elella et al. (2021); Khoshnood et al. (2019); Regnat et al. (2018); Sarubbo et al. (2022); Singh et al. (2020)
	Risk prevention						
		Biologicals/chemicals	Product containing microbial cells or their metabolites for controlling plant disease	Galltrol, Serenade, AtEze, BlightBan, Bio-Save	Commercial formulation, biocontrol, antagonism, competitive exclusion, secondary metabolite, antibiosis, competition, parasitism, and lytic enzymes	Anderson et al. (2004); Lindow et al. (1996)	Hale et al. (2014); He et al. (2021); Keswani et al. (2016); Lahlali et al. (2022)
Biomonitoring			Tracking the microbiome to identify or predict environmental-specific risks		Community structure, community function, succession, and colonization	Aydogan et al. (2020); Chen et al. (2020)	Chandran et al. (2020); Trivedi et al. (2022)
	Risk detection						
		Food safety	Monitor microbial community profiles to designate healthy or non-contaminated plants as well as forecast conditions adequate for human pathogen colonization of food products		Plant pathogens, human pathogens, outbreak, surveillance, and food security	Brandl et al. (2023); Potnis et al. (2014); Williams et al. (2013)	Marshall et al. (2020)
		Microbial forensics	Investigative method for pathogen detection in national and international trade and agricultural biosecurity		Epidemiological mapping, biosecurity, biosafety, inspection, and tracking	Marine et al. (2015); Ottesen et al. (2019)	Doyle et al. (2017); Fletcher et al. (2020); Ochoa-Corona (2011); Schmedes and Budowle (2019)

## Biosupplementing for plant health

“Plant probiotics” is an umbrella term for microorganisms that offer some defined benefit to their plant host (Berlec 2012). Well-known examples of plant probiotics are the so-called plant-growth-promoting rhizobacteria (PGPRs), of which formulations are sold commercially as single or mixed strain products (Kumari et al. 2019). The concept of plant-growth-promoting phyllobacteria (PGPPs) is not as developed as that of PGPRs (Orozco-Mosqueda et al. 2021), but there are many ways in which phyllosphere microbes have been shown to contribute to plant health (Stone et al. 2018). The ability to fix atmospheric nitrogen on the leaf surface is one of them (Abadi et al. 2021; Li et al. 2019b). Under natural conditions, this ability is particularly relevant in tropical rainforest systems where the lack of seasonal leaf senescence and poor soil conditions rely heavily on bacterial-leaf N_2_ fixation (Goncalves et al. 2014; Stanton et al. 2019). The foliar application of nitrogen-fixing bacteria onto agriculturally significant crops has shown increased nitrogen uptake and a reduced need for nitrogen fertilizer input (Abadi et al. 2021; Madhaiyan et al. 2015). Other proposed mechanisms of plant growth stimulation by phyllobacteria that occur naturally but have potential biotechnological utilization are phosphorus solubilization, siderophore production, and the secretion of hormones such as IAA (Abadi et al. 2020; Arun et al. 2020; Berg and Koskella 2018; Cernava et al. 2019; Chacón et al. 2022). Phyllosphere bacteria and fungi also have been shown to alleviate drought stress, for example in rice seedlings (Abadi et al. 2020; Arun et al. 2020; Cernava et al. 2019; Chacón et al. 2022) and panic grass (Aimone et al. 2023).

A major line of research on how to protect plants from foliar diseases involves the isolation and characterization of so-called biological control agents (BCAs). These BCAs antagonize pathogens or pests through a variety of mechanisms including direct antagonism (parasitism and predation), mixed-path antagonism (production of antibiotics, lytic enzymes, waste products, and chemical interference), or indirect antagonism (competition for limited resources and induction of host plant resistance) (He et al. 2021). Commercialized formulations of both fungal and bacterial antagonists exist (Keswani et al. 2016; Lahlali et al. 2022). One of the classical success stories of phyllosphere-derived biocontrol of plant disease is a product that is registered under the name BlightBan A506® that contains, as the active ingredient, *Pseudomonas fluorescens* A506, a bacterial isolate from a pear tree in Healdsburg, CA (Wilson and Lindow 1993). A506 has been shown to protect plants from pathogen-induced frost damage (Lindow et al. 1996) and fruit russeting (Lindow et al. 1998) and from symptoms associated with bacterial fire blight caused by *Erwinia amylovora* (NuFarm 2024). The mechanisms underlying these activities include preemptive exclusion and antibiotic production (Anderson et al. 2004; Temple et al. 2004). Although registration has expired for its use, BlightBan C9-1® was another commercial product for use against fire blight and is derived from the phyllosphere isolate *Pantoea vagans* C9-1. Interestingly, mixtures of the two BlightBan strains did not provide greater protection against fire blight, because *P. fluorescens* A506 produces an extracellular protease that inactivates the antibiotic production in *P. vagans* C9-1 (Anderson et al. 2004).

In the field of biocontrol research, there is a growing interest in using mixtures of microorganisms with plant-protective properties. For example, a cocktail of endophytic bacterial wheat isolates prevented fungal spore germination and protected wheat seedlings from wheat stripe rust caused by *Puccinia striiformis *f. sp*. tritici*, both in greenhouse and semi-field conditions (Kiani et al. 2021). Another study found that microbial consortia consisting of bacteria and fungi displayed broad-spectrum hindrance of foliar phytopathogens as a result of their combined broad gene functionality (Minchev et al. 2021). Although it is challenging to register products containing more than one microbial strain (Keswani et al. 2016), ongoing research will likely continue to focus on the compatibility and synergy of BCAs and their modes of action in pursuit of improved control of foliar pathogens (Hawkes et al. 2021; Li et al. 2022). An exciting recent development is the demonstration that protection against foliar disease may be achieved as an emergent property of whole microbial communities (Berg and Koskella 2018) and those disease-suppressive microbial communities may be developed through passaging of leaf microbiota from one generation of plant leaves to the next, in the presence of a foliar pathogen and by selection for low disease phenotype after each passage (Ehau-Taumaunu and Hockett 2023).

## Phyllosphere microbiology and human health

Phyllosphere-associated microbial communities play a role not only in the health of plants but also in that of humans who consume plant vegetation, for example, as leafy greens. Unfortunately, consumption of fresh produce carries an increased risk of foodborne diseases due to incomplete removal or inactivation of microorganisms that are pathogenic to humans, for example, norovirus, *E. coli*, *Salmonella*, and *Cyclospora* (Machado-Moreira et al. 2019). However, not all microorganisms on plant foliage are harmful to human consumers. In fact, the concept of the “edible microbiome” is based on the idea that with the ingestion of raw plant foods; we load our gut with microbes that associate with these foods that might have probiotic qualities (Berg et al. 2014a; Soto-Giron et al. 2021). Depending on the type of vegetables consumed, and where and how they are grown, humans are exposed to a variety of different microorganisms on and in their food (Leff and Fierer 2013; Marco et al. 2022; Rook 2013). Of special interest are the lactic acid bacteria (LABs) that are commonly found on leaf surfaces (Yu et al. 2020). Many of these LABs confer health benefits to humans. For example, the plant-associated bacterium *Lactiplantibacillus plantarum* (formerly *Lactobacillus plantarum*) can survive gastrointestinal-like conditions, break down food compounds that its human host cannot, antagonize human pathogens, and persist via adherence to epithelial cells (Vitali et al. 2012). Further study into the edible microbiome may uncover additional probiotic strains that could change how we choose or grow our foods, or that serve as the basis for new probiotic products deriving from the phyllosphere which could have an impact on human health and even thwart off human pathogens (Ercolini and Fogliano 2018; Wicaksono et al. 2023b).

Another way in which phyllosphere microbes may benefit human health is by stimulating the immune system. This has been referred to as “natural vaccination” (Berg et al. 2014b) which works by eliciting prophylactic and therapeutic responses in the host. In one study, the microbiota of raw *Brassica* vegetables were sequenced and screened for isolates that were able to produce an enzyme linked to cancer prevention (Wassermann et al. 2017). Enriched among these were members of the *Enterobacteriaceae* family. Interestingly, this family represents a major group in the loading of the gut microbiome through the consumption of fresh produce (Erlacher et al. 2015; Rastogi et al. 2012). Arguably, these bacteria represent a long-established part of the human diet and studies have shown that foodborne microbes that accompany plant-based diets can indeed seed the human gut (David et al. 2014; Wicaksono et al. 2023a).

Fermented foods represent another health benefit related to phyllosphere microorganisms (Marco et al. 2017). A growing body of knowledge suggests that the consumption of live microbes associated with fermented foods can alter the gut microbiome (Roselli et al. 2021). Fermentation into foods such as sauerkraut and kimchi typically depends on the microbes that naturally occur on plant leaf surfaces. *Lactobacillus* species play an important role in the final stages of the fermentation process, and these strains have been shown to have effects on insulin sensitivity (Zhong et al. 2021), Alzheimer’s disease (Kumar et al. 2022) and have anti-cancer (Eweys et al. 2022), anti-mutagenic (Mazanko et al. 2022), immune function (Rastogi and Singh 2022) and anti-obesity (Zhu et al. 2019) properties. We are just beginning to understand the importance of the edible leaf microbiome (Leeming et al. 2021; Tomás-Barberán and Rodríguez 2022), whether raw or fermented and further research will be necessary to unravel how diet and health are related to the incorporation of phyllosphere microbes.

## Bioprocessing for environmental health

Phylloremediation refers to the ability of leaf-surface bacteria to degrade airborne or leaf-surface pollutants (Wei et al. 2017). Examples of these are polycyclic aromatic hydrocarbons that build up from deposition of urban area smog (Ali et al. 2015; Gandolfi et al. 2017), residual pesticides that persist as a result of overuse (Katsoula et al. 2020; Kucharska et al. 2020; Scheublin and Leveau 2013), micro- and nano-plastics (Adomako and Yu 2023), or climate-active gases (Bringel and Couee 2015; Crombie et al. 2018). Several studies suggest that the process of phylloremediation may be harnessed by inoculating leaves with plant-associated pollutant-degrading microorganisms (Weyens et al. 2015). Bacteria that degrade phenanthrene (Dharmasiri et al. 2023) or xylene (Sangthong et al. 2016) and fungi that break down aromatic hydrocarbons (Imperato et al. 2021) have successfully been isolated from leaf surfaces. Levels of hydrocarbon pyrene deposits were significantly reduced on jungle geranium leaves after artificially inflating native populations of phyllosphere isolate *Kocuria* sp. IC3, while the application of a biosurfactant reduced these levels even further (Siriratruengsuk et al. 2017). Similarly, the application of native bacterial degraders of aromatic organic pollutants was shown to reduce aerial concentrations of these compounds (Jindachot et al. 2018). The observation that phenol degradation genes were induced in bacteria from the common phyllosphere genus *Arthrobacter* during leaf colonization, even in the absence of phenol (Scheublin et al. 2014), suggests that phyllosphere microbiota harbor an untapped and primed capacity to destroy unwanted pollutants and/or chemicals. The future challenge lies in finding out how to expand these efforts of “phylloremediation” to achieve the same effects on a larger scale, e.g., near cities where industrial and vehicle emissions are high (Molina et al. 2021). Phyllosphere bacteria have been shown to degrade phthalates, a common plasticizer in agricultural films. When a phthalate-degrading *Rhodococcus* isolate from activated sludge was applied onto vegetables grown in plastic filmed greenhouses, the bacterium readily established and foliar accumulation of phthalates from the air was reduced (Zeng et al. 2022). Bacterial degradation of phthalates has also been documented in rice crops where a native *Bacillus* species was not only capable of degradation, but following its inoculation also shifted the endosphere community towards increased phthalate biodegradation (Liu et al. 2023). The real-world implication of using these types of plastic-degrading isolates or communities is far-reaching, from environmental cleanup to minimizing the consumption of microplastics in our food.

To relieve our dependence on oil and to reduce atmospheric emissions of air pollutants, the search for renewable alternative fuel sources focuses on microbes that can transform plant lignocellulose waste into useful biofuels (Rai et al. 2022). Many epiphytes harbor enzymes for the production of ethanol and butanol (Amadi et al. 2020; Minty et al. 2013), biodiesel or lipid-based fuels (Miao et al. 2020), or methane and hydrogen (Miftah et al. 2022). Hydrogen fuel is of interest because it is carbon-free and produces minimal pollution (Sen et al. 2016). Among the naturally occurring microbes of wheat straw that produce hydrogen, *Lactobacillus* plays a major role in lignocellulose conversion (Ayala-Campos et al. 2022). Wheat straw microbes outperformed cow manure-, ruminal fluid-, and anaerobic sludge-associated microbes in producing hydrogen, revealing plant materials to be an excellent substrate for manufacturing bioenergy (Pérez-Rangel et al. 2015; Valdez-Vazquez et al. 2017). Additionally, the phyllosphere microbiota of crops used specifically for bioenergy has been well-studied for its capacity to benefit host plants (i.e., aid in nutrient acquisition), defend against pathogens, and mitigate drought or salt stresses (Zhalnina et al. 2021). The microbiome of bioenergy crops such as switchgrass and miscanthus has been tracked over seasons to reveal useful functions and core microbes to supply a foundation for future manipulation to maximize yields (Grady et al. 2019; Howe et al. 2023).

## Bioprospecting the leaf microbiome

Bioprospecting is the search for genes, natural compounds, and whole organisms in nature that have commercial economic value (Müller et al. 2016a). Perhaps the best-documented example of bioprospecting in the context of phyllosphere microbiology is the isolation and application of microorganisms for the management of foliar diseases and pests, for instance, the aforementioned BlightBan A506. Many other examples exist of industrial products or applications that have their origin within the leaf-associated microbiota and are produced commercially and at-scale for use in markets as diverse as cosmetics, food, pharmaceutics, and textiles (Müller et al. 2016a). One is xanthan gum, which is sold under the name Keltro® or Xantural®, and is used as a thickening agent for stabilizing emulsified suspensions (CPKelco 2024). The bacterium that produces this compound naturally is the leaf-colonizing pathogen *Xanthomonas campestris*, a causative agent of bacterial leaf spots on brassicas worldwide. Xanthan is a virulence factor in this organism, as it plays a significant role in biofilm formation, producing a hydrated, protected environment during leaf colonization (Bianco et al. 2016). For the production of this economically important polymer, which is used in foods, household cleaners, pesticides, paints, cosmetics, pharmaceuticals, textile dyes, and petrol recovery and production, cultures of *X. campestris* are grown in large bioreactors (Elella et al. 2021). Another phyllosphere-based product is erythritol which is widely used as a zero-calorie sugar substitute but is also an additive in some pharmaceuticals (Rzechonek et al. 2018). The biosynthetic pathway for producing erythritol is widespread among osmophilic phyllosphere bacteria, yeast, and fungi, including members of *Pseudozyma* sp., *Aureobasidium* sp., *Aspergillus nidulans*, Ustilaginomycetes, and the lactic acid bacteria *Leuconostoc oenos*, *L. plantarum*, and *Acetobacter xylinum* (Monedero et al. 2010; Moon et al. 2010). Often, the erythritol biosynthetic pathway genes from these microbes are cloned into organisms that are more easily managed for the production of this sweetener (Regnat et al. 2018).

Biosurfactants are biological molecules that have both hydrophobic and hydrophilic qualities that allow for reduced surface tension. This property is useful for solubilizing compounds and creating emulsifications. Many types of biosurfactants are produced by microorganisms including those that are members of the microbial communities that associate with plant leaf surfaces. Bacterial biosurfactants are cheaper and easier to produce than their synthetic chemical counterparts. Pathways from leaf-dwelling bacteria such as *Arthrobacter* sp., *Pseudomonas* sp., *Acinetobacter* sp., and *Bacillus* sp. are utilized for petrol recovery, foods, pharmaceuticals, and cosmetics (Campos et al. 2013; Varvaresou and Iakovou 2015). Dozens of companies market biosurfactants for various uses in what is a multibillion-dollar industry (Sarubbo et al. 2022).

Other phyllosphere-derived industrial products involve enzymes and other proteins. Phytases break down phytate, which is the main form of phosphate storage in plants. These enzymes are commonly found to be produced by plant-associated microbes including *Aspergillus* sp., *Pseudomonas* sp., *Serratia* sp., and *Bacillus* sp. (Singh et al. 2020). They are marketed under the names Finase®, Natuphos®, and Allzyme® and sold commercially to ranchers worldwide as an amendment to feed for monogastric animals to better utilize phosphorus (Handa et al. 2020). Another enzyme of biotech interest is asparaginase which is used as an anti-cancer treatment by denying asparagine to growing tumor cells. Asparaginase from *E. coli* is marketed under different trade names, but some patients are intolerant to this enzyme, which is why, instead, wider use of the one from the phyllosphere colonizer (and foliar pathogen) *Erwinia chrysanthemi* is employed (Emadi et al. 2018; Salzer et al. 2014), which is marketed under the name Erwinaze (Keating 2013). Another therapeutic drug derived from a phyllosphere microorganism is the antibiotic mupirocin, produced by *P. fluorescens* (Khoshnood et al. 2019). It is used for topical treatment against methicillin-resistant *Staphylococcus aureus* and is prescribed under the name Bactroban® (GlaxoSmithKline 2024). The ice-nucleating activity of *P. syringae* bacteria has been harnessed and turned into technologies that allow altering weather by cloud seeding, snow-making at ski resorts, and freezing foods (Baloh et al. 2019; Pokharel et al. 2021; Wex et al. 2015). SnoMax® has been one of the most successful products based on *P. syringae*’s ice-nucleating protein and has been in use since 1987 for the production of artificial snow (Telemet 2024). Phyllosphere yeasts have been shown to contribute to the degradation of biodegradable plastics used in compost bags, mulches, and other agricultural supplies (Watanabe et al. 2014). These plastics are made from molecules similar to the cutin monomer found in plant cell walls which are the target of degradation by enzymes produced by certain phyllosphere inhabitants (Kitamoto et al. 2011; Saika et al. 2019). Enzymes and microbes with such activities represent an integral part of so-called life-cycle-engineered plastics (García-Depraect et al. 2021).

Phyllosphere microorganisms have long been known to participate in carbon cycling by contributing to the decomposition of leaf litter (Fanin et al. 2021). The process of decomposition can be harnessed with relatively little human intervention to produce compost and silage. Composting is the controlled biodegradation of organic materials, including leaves and other plant waste, whereas animal silage is preserved forage that is used as animal feed during the dry season. For both compost and silage, the required fermentation is carried out by members of the microbial community that is naturally associated with the plant material (Kraut-Cohen et al. 2016). Foliar microbes also play a role during the curing of tobacco and the retting of hemp for fiber production (Law et al. 2016, 2020; Zhou et al. 2021, 2020). Microbial succession is important for these processes, and the enzymatic activity that facilitates this degradation largely derives from leaf-associated microbes (Alper and Stephanopoulos 2009). Based on these examples, the phyllosphere presents an ideal environment from which to isolate microorganisms with applicable potential for biomass conversion of raw plant materials or agricultural waste into useful substances.

In recent years, the development of culture-independent, DNA-based methods has allowed (meta)genomic mining of diverse microbial environments, including the phyllosphere, for novel functions (Khoiri et al. 2021; Methe et al. 2020; Mishra et al. 2021). To give just one example of the power of such an approach is the study by Helfrich et al. (2018) who sequenced the genomes of 224 bacterial strains isolated from *Arabidopsis* leaves to reveal more than 1000 putative natural product biosynthetic gene clusters, many predicted to code for completely novel types of compounds. In mining the phyllosphere microbiota by metagenomics, it benefits to cast the net as wide as possible and take full advantage of the variation that is naturally present in leaf microbial communities due to geography (i.e., location, growing conditions, and season) and plant genotype (Abdelfattah et al. 2021; Miura et al. 2019). Such an approach also may lead to new strategies that rely on engineered plant genotypes and/or artificially imposed environments to select microorganisms with useful functions (Hale et al. 2014; Singh et al. 2021).

## Biomonitoring the leaf microbiota

Phyllobiomonitoring is defined here as the interrogation of leaf surface microbiota for information that helps with the assessment of plant leaf-associated risks. This idea may be applied to several different fields, including agriculture, food safety, and biosecurity. Typically, biomonitoring involves metagenomics to establish the normal operating range of leaf-associated communities in a greenhouse, field, or grove and to interpret deviations from the normal operating range as a potential signal for a change in the environment or physiology of the plant (Burke et al. 2011; Chandran et al. 2020). These analyses may uncover different states of what constitutes a healthy plant leaf microbiota (Müller et al. 2016c; Vorholt et al. 2017) or whether plants are experiencing biotic or abiotic stresses (Chen et al. 2020; Saleem et al. 2017; Yin et al. 2018). There may be good reason to consider phyllosphere communities as sentinels for changes on a global scale. For example, climate disruptions have been shown to cause perturbations in phyllosphere microbial communities, in turn influencing host plant survival, fitness, ecology, and susceptibility to foliar disease (Perreault and Laforest-Lapointe 2021; Rosado et al. 2018; Trivedi et al. 2022; Zhu et al. 2021). Global warming has a demonstrable effect on the structure of bacterial (Aydogan et al. 2020) and fungal (Faticov et al. 2021) phyllosphere community structure, as does drought (Debray et al. 2021), suggesting a role for phyllobiomonitoring in collecting and analyzing data that can be fed into predictive models for climate warning systems.

Similar surveillance approaches may be applied to the field of food safety to alert growers or processors on changes in the microbiota of their crops which correlate with a higher probability of plant or human pathogens (Williams and Marco 2014). The need to monitor for human pathogen outbreaks has increased dramatically; as diets have shifted, demand for raw produce has gone up, and the number of reported cases of enteric disease resulting from the ingestion of contaminated fresh produce has risen, in particular, leafy greens since the 1990s (Marshall et al. 2020). Whereas sources of contamination and duration of persistence of major enteric human pathogens such as *Salmonella enterica* and *E. coli* O157:H7 on leafy greens have been studied extensively, it appears that these human pathogens, which are actually poor leaf colonizers, can benefit from the presence and activity of naturally present microorganisms on the leaf surface, both pre-harvest (Brandl et al. 2023; Cooley et al. 2006; Potnis et al. 2014) and post-harvest (Williams et al. 2013). Culture-independent community profiling to monitor for such facilitators of pathogen establishment would be a great asset to the produce industry.

Microbial forensics utilizes microbiological knowledge and methods to provide evidence in unlawful matters (Fletcher et al. 2020; Schmedes et al. 2016). Generally, it concerns the abuse of pathogenic organisms and/or their metabolites to cause social or economic damage. This field has become more and more valuable as increased travel and trade within or between countries have led to greater opportunities for the transport and introduction of non-native pathogenic microorganisms (Ochoa-Corona 2011). Current biosecurity tools for monitoring agricultural products have addressable gaps in standardization and validation during all precautionary steps from initial assessment and containment to control of the pathogen (Schmedes and Budowle 2019). This threat to food security can occur at any point in the field-to-table chain, from field to postharvest on domestic and imported goods. Phyllobiomonitoring may directly target one or more pathogens at any point in this series. This has already been implicated in tracking sources of food-borne outbreaks of human pathogens (Marine et al. 2015; Ottesen et al. 2019). Tracing bacterial community profiles at each step of the food processing process could enhance agricultural biosecurity and give concrete evidence at what step contamination occurred (Doyle et al. 2017). Snapshots of community profiles could be uploaded to databases that include what a “normal” microbiome looks like from crops grown in specific locations and seasons throughout their processing. Technologies regarding new detection or tracking of phyllosphere microbes will offer safety checks in the chain of food handling and agricultural trade.

## Concluding remarks

Applied microbiology of the phyllosphere is an exciting area of research and development. Its potential is not bound by source material: for example, the total number of bacterial cells estimated to be associated with all of Earth’s combined terrestrial plant foliage is 10^26^ (Lindow and Brandl 2003). This number dwarfs by many orders of magnitude the number of bacterial (and other microbial) cells that have ever been cultured, assayed, and/or sequenced in labs around the world to produce the cumulative body of knowledge that currently exists on the topic of phyllosphere microbiology, of which only the tiniest fraction is covered in this review. The 10^26^ number is a powerful reminder of the vast functional diversity that is contained in the phyllosphere microbiota that we still know very little about. As the examples in this review make clear, the diversity, and the many agricultural, biotechnological, and other products, applications, and services that emerge from it, touch on many facets of life on this planet. Applied phyllosphere microbiology has a proven impact and future promise to enhance the health and productivity of plants, humans, and the environment.
